# Medical student learning on a distributed training platform in rural district hospitals

**DOI:** 10.4102/phcfm.v16i1.4539

**Published:** 2024-08-26

**Authors:** Andrew J. Ross, Thandaza C. Nkabinde, Bernhard Gaede

**Affiliations:** 1 Department of Family Medicine, Faculty of Health Sciences, University of KwaZulu-Natal, Durban, South Africa

**Keywords:** student learning, distributed platform, transformative learning, disorientating dilemmas

## Abstract

**Background:**

Decentralising medical school training enhances curriculum relevance, exposing students to generalist patient care in diverse contexts.

**Aim:**

The aim of the study was to understand the student experiences of learning during their 7-week Family Medicine rural rotation.

**Setting:**

Final year medical students who had completed their Family Medicine rotation in November 2022.

**Methods:**

A qualitative study involving 24 final year students (four semi- structured interviews and four focus group discussions [4 x 5 students]). All interviews were recorded, transcribed verbatim and analysed thematically.

**Results:**

Analysis revealed positive learning experiences and identified the following themes: taking responsibility for learning, the generalist context, teaching and learning in context and managing the learning environment.

**Conclusion:**

Active participation in hospital activities, exposure to disorientating dilemmas that challenged assumptions and reflection on these experiences led to transformative learning and knowledge co-construction.

**Contribution:**

The study contributes to the discussion and reinforces the advantages of distributed, experiential training, highlighting the positive impact of meaningful participation and transformative learning opportunities.

## Introduction

For several years, there has been an increasing trend of decentralising clinical training of medical students from tertiary health care centres, to urban, peri-urban and rural sites.^1,2^ This shift has been influenced by the increasing number of medical students at South African medical schools who cannot be accommodated in tertiary institutions, the need to make the curriculum more relevant to the needs of the country and a recognition of the academic value of students learning in more rural platforms, where they get exposure to generalist care of patients presenting with undifferentiated problems as well as an understanding of the patient context.^3,4,5^ This process of decentralisation of training is not simple as there are multiple factors that need to be considered. Fortunately, a framework for distributed health professionals training was developed in 2015, which helps to guide the implementation of this training.^2^ Although decentralised training is often used to describe training outside of tertiary academic complexes, ‘distributed platform’ as used in this article is seen as a more open, non-hierarchical term.^2^ Distributed training has been described as ‘training activities for undergraduate students that takes place away from tertiary academic complexes’.^6^

Distributed placements are excellent in preparing students for future practice as they develop self-confidence and competencies as they build on preexisting knowledge and skills gained in earlier years.^4^ This self-confidence and competency are achieved through the academic concept of experiential learning, which is the ability to construct knowledge and meaning from real-life experiences encountered in daily practice at distributed sites.^7^ The experiential learning theories provide explanations for how individuals learn in unique ways as they react to their perceptions of experience.^7^ Through these experiences, medical students are stimulated to consider how they can be a self-regulated learner, a concept defined as a process that helps guide an individual’s goal-directed activities by controlling and managing their cognition, affect and behaviour.^8^ It is critical that they have this understanding as upon graduating, they will be expected to function with some level of independence although (initially) still under supervision, and provide optimal health care, while able to identify and willingly engage in ongoing professional development activities that serve to maintain their learning and competence.^9,10^

For students to develop academically as they engage with experiential learning, they must take active responsibility for their learning. This means that they must have a sense of agency for self-directed learning, which ideally should have already been developed prior to entering clinical rotations.^11^ The centrality of responsibility underpins experiential learning, but requires maturity and deep reflection from the learner on their prior experiences in order to fully engage in the present learning opportunities. This ensures transformation of the learner as they interact with authentic ‘real’ scenarios.

### Educational setting/learning environment

The Family Medicine department at the University of KwaZulu Natal (UKZN) believes that exposing medical students to a distributed rural learning environment is critical, as: (1) the rural context provides different but important learning opportunities to those provided in urban teaching hospitals and (2) it is a critical area in which students (and graduates) need to learn to work in. Evidence from around the world suggests that such exposure can influence where graduates decide to practice when their training is complete.^12^ It is important to note that approximately half of the global population live in rural areas and are served by less than a quarter of the world’s medical doctors, with sub-Saharan Africa served by only 4% of the global health workforce.^13^ To address these challenges, there is an urgent need to change selection of students, training content and context and continual support of medical doctors, which in turn would hopefully improve retention of the personnel once placed in such marginalised communities. The Family Medicine rural block is an attempt to expand the context of where training occurs.

The Family Medicine Integrated Primary Care 3 (IPC3) module at UKZN is one of six final year modules that the 6th year medical students have to complete in order to attain their MBChB degree. This module is designed around the CanMEDS framework of core competencies, with assignments organised thematically around key roles of a physician namely: communicator, collaborator, manager, health advocate, scholar and professional. The purpose of the rotation is for the students to experience and to practise primary care medicine that is responsive to patients, their families and the community within the context of a district health system. This module builds on skills and experiences from other family medicine modules done in preceding years, thus allowing them to practically implement their knowledge and skills in a context of a rural district health system, which exposes them to the undifferentiated patients, forcing them to understand where their patient comes from, why they are there, and what their expectations are. Part of the design of the final year Family Medicine module is the placement of students in environments which they are not familiar with educationally. These environments require agency as students have to ‘step up’ and become ‘doctors’ by taking responsibility for patient care, under the supervision of the medical officers.

The students are supervised by medical officers, primary healthcare nurses and members of the interprofessional team during their rotation. These levels of supervision challenge the students’ thinking and norms, as they are used to being supervised and taught by specialists mainly in regional hospitals. Working and living within the same rural environment also allows the students to be exposed to the cultural context, which adds further dimensions to their learning that go beyond the basic clinical learning.

Research suggests that for medical doctors to achieve positive patient outcomes through providing quality patient care, they need a supportive learning environment that fosters research and an evidence-based approach to their work.^14^ The rural district hospitals (DHs) where the final year medical students rotate were selected for student rotations as they provide quality care for the local population, are (reasonably) well-staffed, are supportive of the UKZN Family Medicine block and have staff who are willing to encourage student participation in the day-to-day activities of the hospital. The Department believes that these approaches create an ideal environment for learning.

The student experience on the distributed rural platform has been framed within a particular context that includes: (1) leadership and governance which direct it, (2) the site which provides permission and opportunity to participate, (3) the community, (4) sufficient capacity of the students, (5) student willingness to engage and participate and (6) the structure of the block.^5^ The Family Medicine department has provided leadership and oversight for the distributed platform, selected sites which support students learning, which have adequate infrastructure, and staff who are willing to invest in the training of the next generation of health care professionals.^5^ Final-year medical students are oriented prior to their allocation to DHs, and regular visits to participating DHs by staff from the Family Medicine department at UKZN ensure that roles and expectations are shared with hospital staff. The design of the module with small numbers of students allocated to suitable sites, reflection on disorientating experiences, clear expectations of participation and engagement, provided the opportunity for transformational experiential learning.

In order to fully understand the continuous impact of rural placement of students, and in order to ensure that their experiences remain positive, it is important to evaluate the programme, thus identifying areas needing improvement.^15^ The aim of the study was to understand student learning through the lens of experiential learning in a rural DH training platform for 6th year medical students who did the Family Medicine IPC3 block from 10th October 2022 to 25th November 2022.

## Research methods and design

Final year MBCHB students at UKZN are placed in groups of 2–6 students at one of 16 rural DHs in KwaZulu Natal (KZN) for their 7-week rural block. This qualitative study was done in November 2022 with final year students when they returned to UKZN after the completion of their Family Medicine rotation. Students were asked to participate in this study, and 24 students consented to participate in four semi-structured interviews (SSIs) and four Focus Group Discussions (FGDs) (4 × 5 students). The FGDs were held face-to-face and the SSIs were held online via Zoom at times that were mutually convenient. All of the SSIs and FGDs were facilitated by AR, a faculty member who has extensive experience in qualitative research and who was not involved in any student teaching in the final year Family Medicine module. Students were asked about their understanding of a learning environment, whether or not there was a learning environment at the DH where they were based (and to give examples), and how this impacted on their learning while based at the DH and how this environment compared to other experiences that they had had at other health institutions. Interviews lasted 45 min–1 h, and were recorded and transcribed verbatim. After repeated reading by all the authors, codes, categories and themes were identified from the data and are presented in the results section. Direct quotes are provided where appropriate, and all data reported have been anonymised.

### Ethical considerations

Ethical approval to conduct this study was obtained from the University of KwaZulu-Natal, Biomedical Research Ethics Committee (No. BREC/00004935/2022) and gatekeeper permission was given by the Registrar of UKZN Dr K.E. Cleland.

## Results

Final year medical students were very positive about the rural rotation as, often for the first time in their medical training, they felt that they were becoming doctors as they participated in the day-to-day activities of the hospital. Despite challenges with Wi-Fi connectivity, shortages of consumables and a lack of infrastructure, students recognised that:

‘[*A*] learning environment is literally anywhere, because any situation can be an opportunity to learn.’ (SSI4, SK, 26/11)

They also recognised some of the factors that contributed to the learning environment (at the DH) were:

‘[*S*]upport, opportunity, and responsibility.’ (SSI2, RH, 28/11)

The themes that emerged were grouped broadly into taking responsibility for learning and students’ learning experiences, generalism and the reality of context, being part of the team – teaching in context, and managing the learning environment – the design of the module.

### Taking responsibility for learning and students’ learning experiences

Learning is a dynamic process that needs active participation. The DHs provided the context but there was a need for students to actively participate in that process – to be willing to learn, to actively participate in the opportunities provided, and be willing to take responsibility for the work that was entrusted to them. In this context, the learning opportunities are:

‘[*D*]ependent on you and what you make of it.’ (FGD2, Student 2, 25/11)

‘… and that students needed to be willing to learn and seek information and ask, as someone who shows enthusiasm encourages the teacher.’ (FGD2, Student 2, 25/11)

Although this responsibility was initially:

‘[*V*]ery daunting because you feel a little overwhelmed.’ (SSI2, RH, 28/11)

A willingness to actively participate helped students realise they knew things and to develop confidence in their abilities:

‘[*I*]t was a little scary at first, but it gave me a little bit more confidence as well. I said, okay, if I’m the person that needs to be doing this, this is part of my job, I’m going to have to do this. And at that moment, I took ownership – it gave me that sense of responsibility now that I get to see the patients, but because they are my responsibility that I needed to also do a really thorough job.’ (SSI2, RH, 28/11)

The taking of responsibility was an important trigger for learning as:

‘[*R*]esponsibility motivates a lot more, without the responsibility there may not be that eagerness or willingness. I think you don’t comprehend the seriousness especially when it comes to medicine, you’re dealing with somebody’s life. If there’s no responsibility, if whatever you do doesn’t matter are you going to take it seriously? So, in that sense it put a lot of it into context, what our actions produce consequences.’ (FGD2, Student 3, 25/11)

‘I think it’s important that we were given the opportunity to manage someone on our own so what our primary plan is what the patient is getting so if that was slightly off, the Doctor would come and adjust it, but it allows us to reflect as well. Okay, this is what the type of Doctor I’m gonna be, this is the management plan that was actually done for this patient, so in the future I will always remember okay this Doctor corrected me by adding this on, so in the future I will never forget that.’ (FGD3, Student 2, 25/11)

The structure of their rotation meant that they had first contact with patients whom they had to assess and develop a management plan for, were given responsibilities for patient care, were exposed to common conditions, and had to discuss patients with the doctors. Supervision was provided to ensure:

‘[*S*]afety netting.’ (FGD2, Student 3, 25/11)

‘… and quality patient care but at a distance.’ (SSI4, SK, 26/11);

so that they could make meaningful decisions about the management of patients. Students felt that:

‘[*Y*]ou had supervision, but the independence gives you that room to learn on your own.’ (FGD3, Student 3, 25/11);

which facilitated learning but ensured that patient safety was never compromised and that they were accountable for the patient care.

When students were encouraged to get involved, given meaningful responsibility, trusted, supervised and felt valued and part of the team, it created a supportive environment for experiential learning to flourish. They felt that their work mattered and that they were making a meaningful contribution. This responsibility and accountability encouraged students to find the answers as:

‘[*W*]hen you’re dealing with a patient, and you don’t know the diagnosis, or the treatment, you go on the internet, or you go on the essential drug list (EDL), look at the treatment, look at the symptoms, okay this is how it presented, this is what it is, this is what I must do.’ (FGD3, Student 2, 25/11)

### Generalism and the reality of context

Students recognised that there were factors in the generalist setting at the hospital, the local clinics and in the community that contributed to their learning. District hospitals are by definition generalist and students found the exposure to people-focussed care transformative as they learned about the clinical, social and contextual factors contributing to illness:

‘I remember my first day in outpatients department (OPD) – the first patient had arthritis. It was like, okay – I got this. I remember my stuff. Second patient was uncontrolled hypertension, third patient had vaginal discharge syndrome (VDS). I was like, Okay, This is starting to get a bit out of hand. And next patient came back for a review of his X-ray. I’m not sure what’s going on. The next patient is chronic diarrhoea you know. You are constantly on alerts. You’re constantly learning all the different aspects of medicine.’ (SSI1, PK, 25/11)

First contact with patients in casualty and OPD was an important stimulus for learning and developing confidence as *we* (would):

‘[*C*]lerk and examine the patient and come up with an assessment and plan for the patient and after that discuss it with the senior doctor. And then they will approve and also add some things if they think we missed some things with the management. In other blocks the management is already there in rural you do it yourself, so you really gain a lot, you really gain a lot of confidence going into internship.’ (SSI3, SK, 28/11)

Not:

‘[*J*]ust needing to push the line, meant that students could go to the Doctor in casualty and ask, “I’m not too sure about this patient.” And that Doctor would actually take time out and come see the patient in OPD.’ (FGD3, Student 1, 25/11)

The students experienced person-centred care (as opposed to disease-centred care) which was holistic as the staff were concerned about the context of the patient (does this patient have electricity at home to keep their insulin safe) and humanized medicine. Staff were treating the patient as a person (FGD2, Student 1, 25/11).

Doing home visits with the Ward Based Outreach Teams exposed students to the socioeconomic realities that patients experience every day and:

‘[*W*]as a big part of my learning. Going out with the mobile clinical and the nurses that was very beneficial, because going into the actual community, we are seeing first-hand what is happening, and that was a big learning environment personally, because I had not seen poverty at that level. You know that there is poverty, but you not experiencing it first hand and the home visits were very eye opening … The one patient that I saw he was staying in an attached room, and there was nothing there, there was no toilet, there nothing besides a bed, and it ended up that he needed emergency care, and we actually took him with us back to the hospital because he needed emergency care.’ (SSI2, RH, 28/11)

‘I mean when patients come to you, you’re viewing the disease process. … [*W*]e can understand why a patient will come in with maybe gastro, and then go back home. And then suddenly they’re returning with the same issue because they don’t have a clean water supply, so they’re obtaining their water from the river, and then they’re washing their clothes in a pond … I’m telling them, you know, to do these things, you know to be hygienic, and you know, you can instruct them from the hospital, but the understanding only comes from when you are out there in the community, and seeing what they actually have. I think that was a big part of my learning.’ (SSI2, RH, 28/11)

To better understand whole person medicine, students were required to visit a traditional healer to explore his/her understanding of health. For many students this was:

‘[*M*]y first time. I needed to find an understanding of (why) patients are going to a traditional healer before coming to the hospital, to understand it and build a relationship. I mean the traditional healer has been there for years and built a relationship with the patient and the patient’s family.’ (FGD3, Student 1, 25/11)

### Being part of the team – Teaching in context

Students learnt the value of:

‘[*W*]orking with the different disciplines (which helped me) gain an understanding of what exactly it is they do.’ (SSI2, RH, 28/11)

Although different professions often work in parallel, interprofessional care has been shown to improve patient outcomes and at the DH students were able to see the value of interprofessional collaboration in the care of patients as:

‘[*T*]here was a lot more team work as well as interprofessional collaboration … I saw interaction between the MDT and doctor. So the doctor would see the patient and the dietitian or whoever would be there would assess the patient and they’d discuss it face to face and then make their notes, come up with a plan.’ (FGD2, Student 3, 25/11)

‘Yeah, it’s just multi-disciplinary, so it was a good environment because every Thursday we had grand round meetings where there would be sharing of information from the radiographers, the physios and the dietitians. So, like there is continuous learning at the weekly meetings.’ (FGD3, Student 4, 25/11)

Staff at the DH were keen and willing to teach, actively encouraged student’s participation, even calling them to see interesting patients – with students remembering:

‘[*T*]he one Doctor at casualty yoh she was teaching us everything, like all the skills. There’s a patient to suture, come, there’s an ICD, come, another [lumbar] puncture, come.’ (FGD3, Student 3, 25/11)

‘The MO on call at casualty with us was very enthusiastic to teach us this new skill. He was very knowledgeable and well experienced, very patient because we were making a lot of mistakes after he demonstrated it like once, but he was very patient and after each mistake he would give us constructive feedback, you know tell us where we’re going wrong, how we can improve, very supportive and complementary when we did do it well.’ (FGD1, Student 6, 25/11)

In addition to the informal clinical teaching, staff mentored them:

‘… noticed gaps and were willing to fill in the gaps that you are lacking.’ (FGD2, Student 1, 25/11);

and provided role models in terms of teachability, continuous learning, professional communication and creating a safe environment in which mistakes could be acknowledged and learnt from:

‘Yes, so they told us, feel free to ask me any questions, (and) if I’m not sure of the answer myself, I will go and check it up, and we will discuss [*it*] tomorrow. So, they were also like open to me asking questions.’ (SSI2, RH, 28/11)

The staff at the hospital created an environment in which students could ask questions and learn without feeling like a failure. The staff had the knowledge without the ego and without the toxicness (FGD2, Student 3, 25/11) which they had experienced at some of the central teaching hospitals. Students felt that:

‘[*T*]he environment allowed us to actually question things a lot more, have these kind of discussions with the doctors and say I’m not sure I don’t know or even advocate for a patient.’ (FGD2, Student 3, 25/11)

Students felt that:

‘[*E*]ven when I made mistakes, I made a lot of mistakes, I wasn’t reprimanded as such, but I was given constructive criticism which was very vital for my growth.’ (FGD3, Student 3, 25/11

‘They created an environment that was safe enough for us to be silly, like ask stupid questions. It was based on the foundation of respect, we felt respected and seen. In Durban we’re treated like we’re just after sharps containers, then there’s us, that’s how I felt. So, there we were seen, we were heard, and it made the environment easy for us to communicate our shortfalls and the gaps in our knowledge and how we wanted to be assisted. So it promoted a culture of reading, it promoted a culture of conversation and I felt that instead of regurgitation of information we were thinking.’ (SSI4, SK, 26/11)

Senior management made a concerted effort to ensure that:

‘[*E*]veryone’s on the same level … doctors aren’t treated any special to nurses so everyone is treated with the same level of respect and I think that contributes also to the way doctors treat or teach us students.’ (FGD2, Student 4, 25/11)

Even when a patient died following a failed resuscitation, there was opportunity to discuss and learn from that experience as:

‘[*T*]hey handled it when I spoke about (the failed resuscitation). They told me that this does happen – they reassured me that you know you need to take this as a learning opportunity because you’re gonna be dealing with this next year – they understood because it’s not the first time that it happened these things occur.’ (FGD2, Student 3, 25/11)

Structured learning activities (CME, journal clubs, morbidity and mortality meetings) at the hospitals meant that students:

‘… benefited a lot from those meetings in the morning because they would present different topics.’ (FGD1, Student 2, 25/11)

Patient presentations and critique of the management meant that:

‘[*I*]f they’ve made any mistakes (they learn) what they should do better, so they were encouraging.’ (FGD1, Student 5, 25/11)

In addition, students were encouraged to participate because:

‘[*S*]tudents … you guys are still fresh with the theory. Just please tell us what the latest thing about this and this. So that was actually quite good. It was a safe space.’ (SSI1, PK, 25/11)

However, this was not the case at every hospital with students recognising that at some hospitals:

‘[*T*]he environment wasn’t very safe or comfortable as the senior doctors were very critical of anyone who did provide any feedback.’ (FGD1, Student 6, 25/11)

This had the effect of stifling discussion and opportunities to learn:

‘[*A*]s seniors are very harsh, they do ridicule you at times.’ (FGD1, Student 6, 25/11)

In addition not all the MO’s were equally willing to spend time teaching students. Although there were opportunities for students to get involved in patient management their experiences was that:

‘[*S*]ometimes the doctors would just suture, they would not teach you that there’s interrupted suture, there’s continuous suture, there’s these type of knots.’ (FGD1, Student 2, 25/11)

### Managing the learning environment – Design of the module

There were important aspects in the module design aimed at maximising the learning opportunities in the environment where students are placed. These included smaller number, identifying suitable trainings sites and communicating clearly the expectations the module would place on students.

University of KwaZulu Natal has identified 16 DHs in KZN where students can do the rural block and place small groups of 2–4 students in each site that is being used. The smaller number of students to medical staff meant that:

‘[*T*]here was *no standing back*.’ (FGD2, Student 3, 25/11)

‘[*T*]his is your opportunity to *shine, your opportunity* to learn. As the teacher to student ratio made a big difference (which meant) that our intakes were one on one, one student with one doctor, so we got to be a lot more hands on, we got to manage a patient from start to finish just with some oversight, some supervision.’ (FGD2, Student 3, 25/11)

‘[*W*]e got the attention we felt we were needed and that pushed me to learning even more.’ (SSI4, SK, 26/11)

Smaller numbers also meant that:

‘[*B*]ecause it was just the two of us the doctors ended up knowing us – so if there was something they’d know, “ah call the students they might like to see this, call the students we have an ascitic tap, call the students.” – I think that allowed us more exposure.’ (FGD4, Student 5, 25/11)

Suitable training sites are essential and it is important to recognise that not all staff are interested or willing to teach. Students at sites where they perceived that they were not valued, where staff suggested that:

‘They could do something better with (their) time.’(FGD1, Student 6, 25/11)

Found the experience:

‘A bit discouraging.’ (FGD1, Student 6, 25/11)

Which had a negative effect on their learning:

‘Yeah, also while I was in the wards I did have a few encounters with doctors who would just tell me – “[*Y*]ou know you don’t have to be here, you can leave you know.” They just didn’t want to have you around. They didn’t see any value in you being present in the wards. So, I would go, I would introduce myself, I would offer to do the work for them, clerk patients, present to them but yeah they weren’t interested they thought that like I could do something better with my time.’ (FGD1, Student 5, 25/11)

Despite willingness of the staff to have students and to teach and supervise students, it is essential that the university communicates clearly to ensure that all hospital staff are aware that students are coming and what students should be involved with. Without good communication students may not be expected:

‘Unfortunately, at XXX, the meetings were there, but for the first three weeks we didn’t get to attend the meetings. The first one they chased us out, and we tried to explain that it is part of our role that we need to attend these meetings, it seems like they are not aware of the structure of our rotation they said no we are not allowed. And our supervisor was on leave at that time, so we had no one. But, when she came back, we discussed it with her, and they actually allowed us in.’ (FGD1, Student 1, 25/11)

The Department of Family Medicine has structured the block around the principles of experiential learning rather than didactic teaching and for most students the learning process did not feel like an academic exercise. Students are used to:

‘[*L*]ectures on a Monday morning, journal clubs on a Wednesday afternoon – we want to be presenting. But when it comes to district hospitals its not academic but focuses on patient management.’ (SSI1, PK, 25/11)

Students also felt that the structure of the programme during the Family Medicine rotation, facilitated their learning. There were weekly assignments that were linked to areas of the learning environment and process that they needed to complete which:

‘[*P*]ushes you that every week you have to study something and on Sunday you had to submit an assignment. So that made us keep on reading, and facilitated learning goals and learning objectives for us the whole week.’ (FGD3, Student 3, 25/11)

## Discussion

The aim of the study was to explore student learning though the lens of experiential learning in a rural DH training platform. While participants recognised that learning opportunities ‘*were everywhere*’ (SSI4, SK, 26/11), recognition of gaps between prior knowledge and the experiences trigged by real life situations was an essential first step in their learning, enhanced by critical reflection of the experiences.^16^ These hands-on experiential learning opportunities stimulated students to recognise their gaps, reflect on these and to construct knowledge (learnings). The assignments of this block require reflection using the what (concrete experience), what now (what was triggered and why and what am I going to do in response to that situation) and so what (which required agency – how can I apply my learning in future situations) format which is a modification of Kolbs experiential learning cycle.^17^ Experiential learning thus relies on (student) engagement, reflection and application of knowledge. Artino et al.^18^ see this reaction to experience as the stimulation to become a self-regulated lifelong learner with goal-directed activities achieved by controlling and managing cognition (thinking), affect (emotion) and behaviour (action). This creates lifelong learners who are able to move from an ‘all knowing’ (traditional view of a doctor) to an ‘all learning’ doctor. Medical educators need to facilitate this by emphasising how students learn rather than how students are taught.^5^

The rural DH context created multiple opportunities for meaningful patient interactions which facilitated student learning about holistic, patient-centred care, social determinants of health and the role context plays in health and illness.^2^ Seeing undifferentiated patients as the first contact health care provider challenged students to be patient-centred (rather than disease centred) and to apply their knowledge in an integrated fashion (rather than siloed) when seeing patients with multiple problems. While still being supervised, students were also expected to take much greater responsibility for engaging with patients and planning their management. These were disorientating experiences as students were used to seeing patients already ‘allocated’ to specific domains and often being observers rather than actors in the management of patients. In addition, home and clinic visits gave them new insights into the context and challenged their assumptions about the validity of the advice they provided, to consider alternative solutions and appreciate the role and contribution of the multi-disciplinary team (MDT) in ensuring comprehensive care. For some students, this was the first real exposure to the social determinants of health (distance to facility, poverty, water issue, etc.), highlighting the complexity of addressing these social determinants, reducing health inequities, and barriers to health access and understanding the critical role of the MDT and a multisectoral approach to these issues, in collaboration with all the stake holders.

By reflecting on their previous understanding, these disorientating dilemmas can lead to transformative learning as baseline beliefs/assumptions are challenged by these encounters as students made new meaning.^19^ This access allowed for the giving, and taking of responsibility for patient care by final-year students, which was a significant stimulus for their learning. Recognition by the students of the many learning opportunities presented, a willingness to take responsibility for their own learning and a need to apply that learning in the context of patient management stimulated authentic learning in keeping with the development as a lifelong learner. This experiential learning, enabled students to build on pre-existing knowledge as they gained competency,^7^ by participating in meaningful and authentic work, done in a supportive environment under appropriate supervision with constructive feedback.

Relationships with hospital staff and a learning environment where students felt valued, respected and heard (no silly questions, we are all learning), that they were able to contribute to service delivery, recognise gaps in their knowledge and take responsibility for their learning from these experiences. Continuity of relationships between key stakeholders, clear roles and responsibilities and trust in the capabilities of students facilitated access and enabled students to integrate into the hospital team and immerse themselves in the hospital context. Meaningful participation in the activities of the hospital created symbiotic, bidirectional and reciprocal relationships, contributed to collaborative learning and co-production of knowledge between patients, students, staff and faculty. The supervision, support, role modelling and mentoring imparted by medical officers were provided because of these relationships.

While efforts to create national consensus around the value of teaching and training on a distributed platform are to be commended,^2^ meaningful relationships with local stakeholders (community, hospital staff, faculty members, students)^2^ are essential as much of the significant learning is mediated through these relationships. These local relationships arising from ongoing interactions among role player are often serendipitous, difficult to achieve yet essential in reaching the expected outcome.^5^ The framework needs modification to show that relationships are all-encompassing and should be placed on the outside of the circle rather than as a small circle in the middle.

The vision for distributed health professions training, which the Family Medicine Department at UKZN has embraced, is that health professional learning should be transformative, reflective, self-directed, inter professional, collaborative and peer-to-peer, socially accountable and community engaged.^2^ Findings from this study provide further evidence of the benefit of training on a distributed platform. These include practical hands-on experiences, exposure to the breath of the health care system in rural and underserved areas, providing generalist care to patients with undifferentiated problems, seeing a broad range of patients in terms of the ecology of medical care, exposure to the burden of disease relevant to the local community, insight into the social determinants of health, working with the MDT, and the possibility of rediscovering their altruism which is often lost at medical school.^5,6,15^

### Limitations

There are inherent limitations to qualitative studies as only a relatively small number of people participated in the study, and the findings cannot be generalised to all students doing the rotation or other settings where a similar module is being implemented. However, the richness of the information obtained provides important insight that deepens our understanding of how students engage in the particular learning environment.

## Conclusion

In conclusion, this study reinforces the advantages of distributed, experiential training, highlighting the positive impact of meaningful participation and transformative learning opportunities. Building long-term relationships with local health care professionals on distributed platforms and structuring learning activities to ensure active participation, which allows students to take responsibility, is essential. Universities are encouraged to provide opportunities for student rotations through distributed training platforms focussing on disorientating experiences that trigger student reflection and development into lifelong learners.

## References

[1] Crampton PES, McLachlan JC, Illing JC. A systematic literature review of undergraduate clinical placements in underserved areas. Med Educ. 2013;47(10):969–978. 10.1111/medu.12215 24016167

[2] Van Schalkwyk SC, Couper ID, Blitz J, De Villiers MR. A framework for distributed health professions training: Using participatory action research to build consensus. BMC Med Educ. 2020;20(1):154. 10.1186/s12909-020-02046-z 32410654 PMC7227246

[3] Green-Thompson LP, McInerney P, Manning DM, Mapukata-Sondzaba N, Chipamaunga S, Maswanganyi T. Reflections of students graduating from a transforming medical curriculum in South Africa: A qualitative study. BMC Med Educ. 2012;12(1):1–9. 10.1186/1472-6920-12-49 22742710 PMC3460748

[4] Mlambo M, Dreyer A, Dube R, Mapukata N, Couper I, Cooke R. Transformation of medical education through decentralised training platforms: A scoping review. Rural Remote Health. 2018;18(1):4337. 10.22605/RRH4337 29522688

[5] De Villiers M, Van Schalkwyk S, Blitz J, et al. Decentralised training for medical students: A scoping review. BMC Med Educ. 2017;17(1):196. 10.1186/s12909-017-1050-9 29121923 PMC5680751

[6] De Villiers MR, Blitz J, Couper I, et al. Decentralised training for medical students: Towards a South African consensus. Afr J Prim Health Care Fam Med. 2017;9(1): e1–e6. 10.4102/phcfm.v9i1.1449 PMC5645562 29041802

[7] Yardley S, Teunissen PW, Dornan T. Experiential learning: Transforming theory into practice. Med Teach. 2012;34(2):161–164. 10.3109/0142159X.2012.643264 22288996

[8] Artino A, Brydges R, Gruppen LD. Self-regulated learning in healthcare profession education: Theoretical perspectives and research methods. In: Cleland J, Durning SJ, editors. Researching medical education. West Sussex: Wiley, 2015; p. 267–278.

[9] Duffy FD, Holmboe ES. Self-assessment in lifelong learning and improving performance in practice physician know thyself. JAMA. 2006;296(9):1137–1139. 10.1001/jama.296.9.1137 16954495

[10] Regehr G, Eva K. Self-assessment, self-direction, and the self-regulating professional. Clin Orthop Relat Res. 2006;449:34–38. 16735869 10.1097/01.blo.0000224027.85732.b2

[11] Richards J, Sweet L, Billett S. Preparing medical students as agentic learners through enhancing student engagement in clinical education. Asia Pac J Coop Educ. 2013;14(4):251–263.

[12] Cancedda C, Farmer PE, Kyamanywa P, et al. Enhancing formal educational and in-service training programs in rural Rwanda: A partnership among the public sector, a nongovernmental organization, and academia. Acad Med. 2014;89(8):1117–1124. 10.1097/ACM.0000000000000376 24979292

[13] Hatcher AM, Onah M, Kornik S, Peacocke J, Reid S. Placement, support, and retention of health professionals: National, cross-sectional findings from medical and dental community service officers in South Africa. Hum Resour Health. 2014;12(1):1–13. 10.1186/1478-4491-12-14 24571826 PMC3975958

[14] Oman KS, Fink RM, Krugman M, Goode CJ, Traditi LK. Rural hospital web-based, evidence-based practice professional development: Challenges and opportunities. J Nurs Profess Dev. 2013;29(2):58–63. 10.1097/NND.0b013e318286c5f4 23657035

[15] Diab P, McNeill PD, Ross AJ. Review of final-year medical students’ rural attachment at district hospitals in KwaZulu-Natal: Student perspectives. S Afr Fam Pract. 2014;56(1):57–62. 10.1080/20786204.2014.10844584

[16] Mezirow J, editor. A transformative theory of adult learning. Adult Education Research Annual Conference Proceedings; 1990 May 18–20; Athens, Georgia: Georgia Centre for Continuing Education; 1993.

[17] Kolb D, Boyatzis R, Mainemelis C. Experiential learning theory: Previous research and new directions. In: Sternberg RJ, Zhang LF, editors. Perspectives on cognitive, learning, and thinking styles. Cleveland, OH and NJ: Lawrence Erlbaum; 2000.

[18] Artino AR, Jr, Gavarkovs A, Brydges R, Gruppen LD. Self-regulated learning in health profession education: Theoretical perspectives and research methods. In: Cleland J, Durney SJ, editors. Researching medical education. New Jersey: Wiley- Blackwell, 2022; p. 267–278.

[19] Mezirow J. Transformative learning as discourse. J Transform Educ. 2003;1(1): 58–63. 10.1177/1541344603252172

